# A 2D nanotheranostic platform based on graphene oxide and phase-change materials for bimodal CT/MR imaging, NIR-activated drug release, and synergistic thermo-chemotherapy

**DOI:** 10.7150/ntno.64790

**Published:** 2022-05-24

**Authors:** Mehri Mirrahimi, Zahra Alamzadeh, Jaber Beik, Abolfazl Sarikhani, Mahdie Mousavi, Rasoul Irajirad, Tahereh Khani, Elnaz S. Davani, Ali Farashahi, Tahereh Shakerian Ardakani, Jeff W.M. Bulte, Habib Ghaznavi, Ali Shakeri-Zadeh

**Affiliations:** 1 Finetech in Medicine Research Center, Iran University of Medical Sciences, Tehran, Iran; 2 Department of Physics, Payame Noor University, Tehran, Iran; 3 The Russell H. Morgan Department of Radiology and Radiological Science, Division of MR Research, The Johns Hopkins University School of Medicine, Baltimore, MD, USA; 4 Cellular Imaging Section and Vascular Biology Program, Institute for Cell Engineering, The Johns Hopkins University School of Medicine, Baltimore, MD, USA; 5 Pharmacology Research Center, Zahedan University of Medical Sciences, Zahedan, Iran

**Keywords:** Graphene oxide, Stimuli-responsive, Phase-change material, Thermo-chemotherapy, Nanotheranostics

## Abstract

Recent years have seen considerable progress in the development of nanomedicine by the advent of 2D nanomaterials serving as ideal platforms to integrate multiple theranostic functions. We synthesized multifunctional stimuli-responsive 2D-based smart nanocomposites (NCs), comprising gold nanoparticles (AuNPs) and superparamagnetic iron oxides (SPIOs) scaffolded within graphene oxide (GO) nanosheets, coated with doxorubicin (DOX)-loaded 1-tetradecanol (TD), and further modified with an alginate (Alg) polymer. TD is a phase-change material (PCM) that confines DOX molecules to the GO surface and melts when the temperature exceeds its melting point (*T*m=39 °C), causing the PCM to release its drug payload. By virtue of their strong near-infrared (NIR) light absorption and high photothermal conversion efficiency, GO nanosheets may enable photothermal therapy (PTT) and activate a phase change to trigger DOX release. Upon NIR irradiation of NCs, a synergistic thermo-chemotherapeutic effect can be obtained by GO-mediated PTT, resulting an accelerated and controllable drug release through the PCM mechanism. The biodistribution of these NCs could also be imaged with computed tomography (CT) and magnetic resonance (MR) imaging in vitro and in vivo. Hence, this multifunctional nanotheranostic platform based on 2D nanomaterials appears a promising candidate for multimodal image-guided cancer therapy.

## Introduction

Two-dimensional (2D) nanomaterials with unique sheet-like structures and physicochemical properties have a wide range of biomedical applications. The ultra-large surface area of 2D nanomaterials can be exploited as a carrier platform for the delivery of functional molecules, including chemotherapeutics 1, 2, genes 3, and proteins 4. Most 2D nanomaterials exhibit strong near-infrared (NIR) light absorption, enabling their use for photothermal therapy (PTT) of cancer 5-12. A variety of functional nanoparticles can be loaded onto the surface of 2D nanomaterials to enrich them for cancer theranostic applications 13-17. Over the last decade, graphene and its derivatives, the most well-studied class of 2D nanomaterials, have been used as a versatile nanoplatform capable of integrating multiple therapeutic and diagnostic functions 18, 19.

Monotherapy of cancer is often associated with side toxicity and limited treatment efficacy due to tumor heterogeneity and the development of drug resistance 20. On the contrary, several recent studies have used combination therapy for obtaining maximum therapeutic benefits caused by the synergistic interaction between the different treatment paradigms while diminishing side effects 21-24. Ideally, a synergistic therapy uses on-demand spatiotemporal delivery of therapeutic agents in response to different stimuli. Graphene-based smart platforms have now become available to perform stimuli-responsive treatment combinations in a synergistic fashion 25-29. This includes controlled release of therapeutic agents in response to either endogenous (e.g., pH 30, glutathione 31, and enzyme 32) or exogenous (e.g., light 33, magnetic field 34, and ultrasound 1) stimuli. In contrast to endogenous stimuli that are difficult to control and have large variations between individuals, exogenous stimuli offer a better means to control on-demand drug release.

One strategy for loading drugs onto the surface of graphene oxide (GO) nanosheets is to use a heat-responsive polymeric matrix. To this end, GO nanosheets can be surface-modified with the phase-change material (PCM) 1-tetradecanol (TD), having a melting point (*T*_m_) of 39 °C, which can be loaded with drugs allowing controlled drug release by increasing the temperature 35. Recent efforts in drug delivery have focused on the design of PCM-based nanosystems in order to control the time frame of drug release. A number of studies have now tested the utility of PCM for stimuli-responsive cargo delivery. For instance, a PCM-based drug delivery system has been developed by filling the hollow interior of gold nanocages with TD as the loading matrix for ultrasound-triggered drug release 36. In addition, TD has served as a gate-keeper to regulate drug release from mesoporous silica-coated gold nanorods upon NIR irradiation 37. Studies on PCM-based drug delivery systems have been mostly restricted to 3D nanostructures that have hollow interiors for drug loading 38. So far, few studies have attempted to integrate sheet-like 2D nanostructures with PCM for on-demand cargo release. In this study, we aimed to employ TD as a PCM to retain doxorubicin (DOX) molecules on the surface of GO nanosheets.

Considering their strong NIR light absorption and photothermal conversion efficiency, GO nanosheets may enable PTT for thermoablation of tumor cells 39. Additionally, the PTT-induced heat generation elevates the local temperature beyond the *T*_m_ of TD, initiating the melting process of TD and subsequent release of payload. Here, NIR activation simultaneously combines thermo-chemotherapy with accelerated drug release. In order to visualize the biodistribution of the graphene-based nanocomposites (NCs) in vivo, gold nanoparticles (AuNPs) and superparamagnetic iron oxide (SPIO) NPs were anchored on GO nanosheet surface to enable computed tomography (CT) 40-43 and magnetic resonance imaging (MRI) 44-46. The surface of the NCs was also modified with an alginate (Alg) polymer hydrogel to improve their biocompatibility and colloidal stability under physiological conditions 47. We show here that these GO-SPIO-Au-DOX-TD-Alg NCs represent a versatile nanotheranostic platform, including synergistic NIR-stimulated thermo-chemotherapy, NIR-triggered drug release, and CT/MR bimodal imaging.

## Experimental section

### Synthesis of GO-SPIO-Au

GO was prepared by oxidizing graphite sheets according to a modified Hummer's protocol 48. The obtained GO product was washed once with 5% HCl, and repeatedly with distilled water to neutralize pH, and then subjected to ultrasonication for 30 min to exfoliate the oxidized graphite and finally was filtered (0.22 μm pore size). Next, 199 mg FeCl_2_.4H_2_O was dissolved in 1.3 mL deionized water under vigorous stirring for 30 min, followed by gradually adding 1.1 mL ammonium hydroxide to the suspension while stirring for another 30 min. The resulting mixture was added to the GO suspension (96 mg GO in 30 mL deionized water) and the sample was heated at 200 °C for 8 h in an autoclave to form GO-SPIO. Then, 0.5 mL of 1 % w/w HAuCl_4_.3H_2_O was added to the GO-SPIO product dispersed in 50 mL deionized water and the solution was heated to boiling temperature. 0.8 mL of 1 % w/w trisodium citrate dihydrate (Na_3_C_6_H_5_O_7_.2H_2_O) solution got warmed (up to 80 C) and then added to the sample and stirred for 5 min. Finally, the mixture was washed repeatedly with deionized water and dried in an oven at 40 C for overnight to obtain the final GO-SPIO-Au product.

### Drug loading and release

TD (0.01%) was placed in a glass vial and melted at 45 °C, followed by the addition of 12 mg/mL DOX upon stirring for 15 min. GO-SPIO-Au dispersed in ethanol was added to the TD/DOX mixture and the temperature was maintained at 45°C to evaporate ethanol. To remove unloaded DOX, the sample was dispersed in cold water to generate two phases between TD and water, one being the TD/DOX mixture and the other DOX-loaded NCs in water. The sample was then centrifuged at 3000 rpm for 15 min and the supernatant was removed. Finally, 0.4% Alg solution was gradually added to the mixture under sonication to obtain a uniform product. Drug-loaded NCs were then placed in a water bath at 37, 41 or 45 °C for 24 h. At predetermined time intervals, samples were centrifuged at 2000 g for 5 min. The supernatants were then collected and the absorbances was measured at 485 nm using a UV-VIS spectrophotometer (Rayleigh UV-1601).

### Physicochemical characterization

NCs were characterized by field emission scanning electron microscopy (FE-SEM; MIRA3-TESCAN) and transmission electron microscopy (TEM; LEO 906; Zeiss). Fourier transform infrared (FTIR) spectra were acquired using a Prestige-21 spectrophotometer (Shimadzu, Japan) equipped with the KBr pellet technique. UV-visible absorption spectra of NCs were recorded using a Rayleigh UV-1601 instrument. The crystalline structure of NCs was investigated with X-ray diffraction (XRD-6000, Shimadzu, Japan). The magnetic properties of the NCs were measured at room temperature using a vibrating sample magnetometer (VSM; MDK6, Kavir, Iran). The photothermal performance of NCs was determined by recording the temperature variation of the NC solution upon NIR irradiation. To this end, varying NC concentrations (0, 25, 50, 100, and 250 µg/mL) were exposed to a continuous-wave 808 nm laser source (Nanobon Company, Tehran, Iran) at a power density of 1.8 W/cm^2^ for 5 min. The temperature was monitored in real-time using an infrared (IR) thermal imaging camera (Testo 875-1i, Germany).

### *In vitro* and *in vivo* CT/MR imaging

For CT imaging, aqueous NC solutions of various concentrations were imaged using a clinical CT scanner (Philips Brilliance 64) operating at 80 KVp and 120 mAs, with a slice thickness=2 mm and a field of view (FOV)=20×10 cm. For MR imaging, NC solutions of varying concentrations were scanned using a clinical MRI scanner (3T, Magnetom Prisma, Siemens, Germany). MR images were acquired with the following parameters: repetition time (TR)=2000 ms; echo time (TE)=12-168 ms with 12 ms increments; slice thickness=2 mm; matrix size=256×256; and FOV=20×10 cm.

To test the bimodal CT/MR contrast enhancement of NCs *in vivo*, CT26 tumor-bearing mice were imaged before and 24 h post intraperitoneal (i.p.) or immediately post intratumoral (i.t.) injection of NCs at **30** mg/kg Au or 5 mg/kg Fe. To quantify the concentration of Au and Fe, NC sample was dissolved in aqua regia under heating (90°C) and analyzed using inductively coupled plasma mass spectrometry (ICP-MS). *In vivo* CT imaging was conducted at 80 KVp and 120 mAs with a slice thickness of 1 mm. *In vivo* 3T MR imaging was performed using a 2-channel phased array 50×16 mm surface coil with the following parameters; turbo spin-echo sequence; TE=10 ms, TR=3650 ms; slice thickness=0.8 mm; matrix size=192×**15**4; and FOV=122 mm. The images were converted into color maps in MATLAB to display changes in signal intensity.

### *In vitro* cell uptake, hemolytic and cytotoxicity assays

The murine colon adenocarcinoma cell line CT26 was cultured in RPMI 1640 medium supplemented with 10% FBS, 100 units/mL penicillin, and 100 µg/mL streptomycin at 37 °C in 5% CO_2_. Cells were incubated with 50 µg/mL NCs for **6** h and then examined with TEM (LEO 906; Zeiss) as previously described 49. To assess cytotoxicity, cells were seeded on 96-well plates at a density of 5×10^3^ per well, and then treated with varying concentrations of NCs (0-100 µg /mL), DOX (0-2 µg/mL) and DOX-NC (NC concentration 0-50 µg/mL, DOX concentration: 0-2 µg/mL). After incubation for **6** h, cells were washed three times with PBS and the cell viability was measured using a 3,[4,5-dimethylthiazol-2- yl]-2,5-diphenyl-tetrazolium bromide (MTT) assay. The hemocompatibility of the NCs was also investigated according to previous studies 50.

### *In vitro* combined thermo-chemotherapy

Cells seeded on 96-well plates at a density of **5** × 10^3^ per well were incubated with DOX and DOX-NC with an equivalent DOX concentration of 0.8 µg/mL. After incubation for 3 h at 37 °C in 5% CO_2_, cell plates were placed in a water bath at 37, 40, and 43 °C for 1 h, and then incubated at 37 °C overnight. To evaluate the combined effect of PTT and chemotherapy, cells were treated with DOX (0.8 µg/mL), NCs (20 µg/mL) and DOX-NC (NC concentration 20 µg/mL, DOX concentration 0.8 µg/mL) for 6 h (37 °C, 5% Co_2_) and then irradiated with NIR at various power densities of 1.2, 1.6, and 1.8 W/cm^2^ for 5 min. An MTT assay was performed 24 h after treatment. Cells were also co-stained with fluorescein diacetate (FDA) and propidium iodide (PI) to visualize live and dead cells, respectively. To this end, 8 µL of FDA dissolved in DMSO (5 mg/mL), 50 µL of PI dissolved in PBS (2 mg/mL) and 5 mL culture medium were mixed and added to cells for 15 min at room temperature, and cells were examined using a fluorescence microscope (Zeiss, Oberkochen, Germany).

An annexin-V-fluorescein isothiocyanate (FITC)/PI assay (eBioscience, USA) was performed to assess apoptosis in CT26 cells in response to the various treatments. Briefly, 24 h the treatment, cells were harvested and resuspended in 1x binding buffer at a density of 1×10^6^ cells/mL. Next, 5 μl of Annexin-V-FITC and 5 μl of PI staining solution were added to 100 μl of cell suspension and the sample was incubated for 15 min at room temperature in the dark. Cell samples were then analyzed by flow cytometry (BD FACSCanto II, USA) for the quantitative measurement of necrosis (PI staining) and apoptosis (annexin-V-FITC staining).

### *In vivo* therapeutic studies

All *in vivo* experiments were conducted in accordance with guidelines established by our Institutional Animal Care Committee. Male Balb/c mice (5-8 weeks old, 20-25 g) were obtained from the Pasteur Institute of Iran. For tumor induction, mice were injected subcutaneously on the right flank with 2×10^6^ CT26 cells diluted in culture medium. When the tumor volume reached approximately 100 mm^3^, animals were randomly divided into five groups (n=5 each): control, DOX, DOX-NC, NC + NIR, and DOX-NC + NIR. For DOX- and DOX-NC-treated groups, mice were injected i.p. with an equivalent DOX concentration of 5 mg/kg. For the groups receiving PTT alone (NC + NIR) and combined thermo-chemotherapy (DOX-NC + NIR), NIR irradiation was performed 24 h post-i.p. injection when the NCs reached an efficient concentration inside the tumor according to the *in vivo* imaging experiments. Mice were exposed with an 808 nm laser source at a power density of 0.7 W/cm^2^ for 15 min. During NIR irradiation, the tumor superficial temperature variation was recorded in real-time using an IR thermal imaging camera. Following treatment, tumor size and bodyweight were recorded for up to 21 days.

### Post-mortem analysis

Tumors were collected from mice on day 3 post-treatment, fixed in 10% neutral buffered formalin, embedded in paraffin, and stained with hematoxylin and eosin (H&E). Vascular damage was assessed by immunohistochemical (IHC) staining using an anti-platelet endothelial cell adhesion molecule-1 (PECAM-1, CD31) polyclonal antibody. To this end, 5 µm thick tissue sections were deparaffinized with xylene, rehydrated with methanol, and then treated with Tris/EDTA buffer (pH=9.0, 10 mM Tris, 1 mM EDTA). A primary antibody against CD31 (cat. E-AB-70021, 1:100, Elabscience^®^) was added and incubated overnight at 4 °C. Tissue sections were washed with PBS and an FITC-conjugated goat anti-rabbit IgG (H+L) secondary antibody (cat. E-AB-1014, 1:100, Elabscience^®^) was added and incubated at room temperature for 2 h. Sections were washed with PBS and cover-slipped with DAPI (4′,6-diamidino-2-phenylindole-2HCl) and then imaged with a fluorescence microscope (Olympus AX70, Japan). H&E and IHC-stained tissue section images were digitized using ImageJ software. To determine NC localization, Prussian blue (PB) staining was performed for the tumor and liver, lung, spleen, kidney and heart, all removed 24 h post-injection of NCs. To this end, 5 μm thick tissue sections were incubated with staining solution containing equal parts of 5% HCl and 5% potassium ferrocyanide for 30 min, and then counterstained with nuclear fast red.

## Results and Discussion

### Physicochemical characterization of NCs

**Figure 1:**
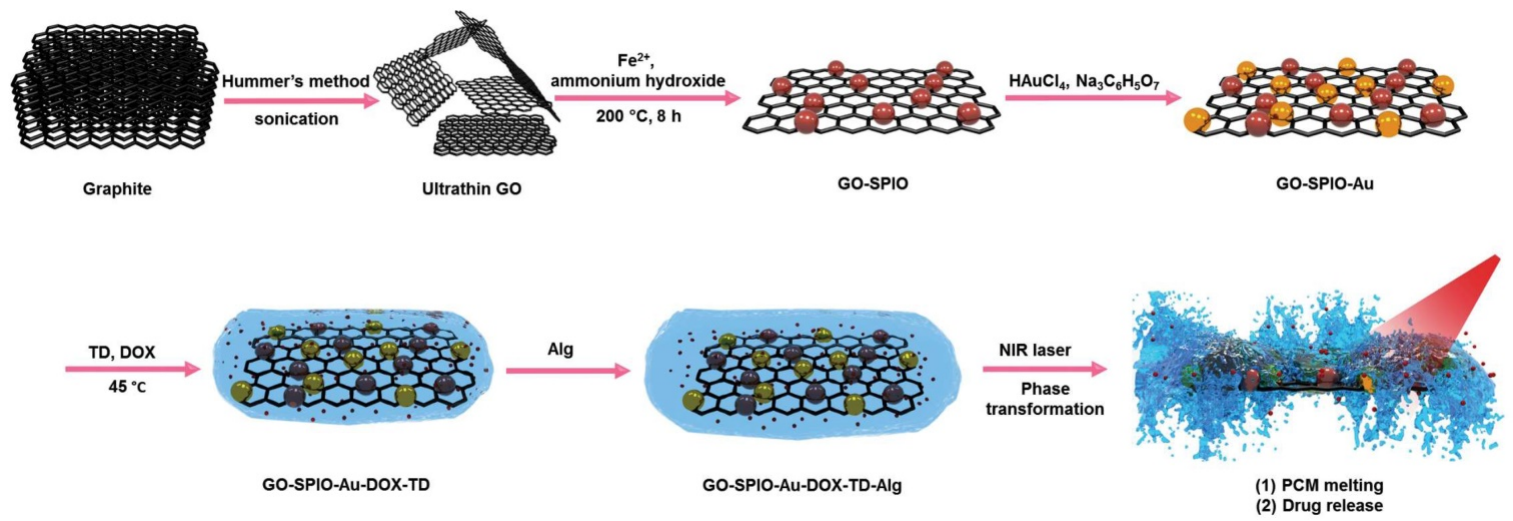
Schematic outline of the different synthesis steps for creating GO-SPIO-Au-DOX-TD-Alg NCs.

**Figure 2:**
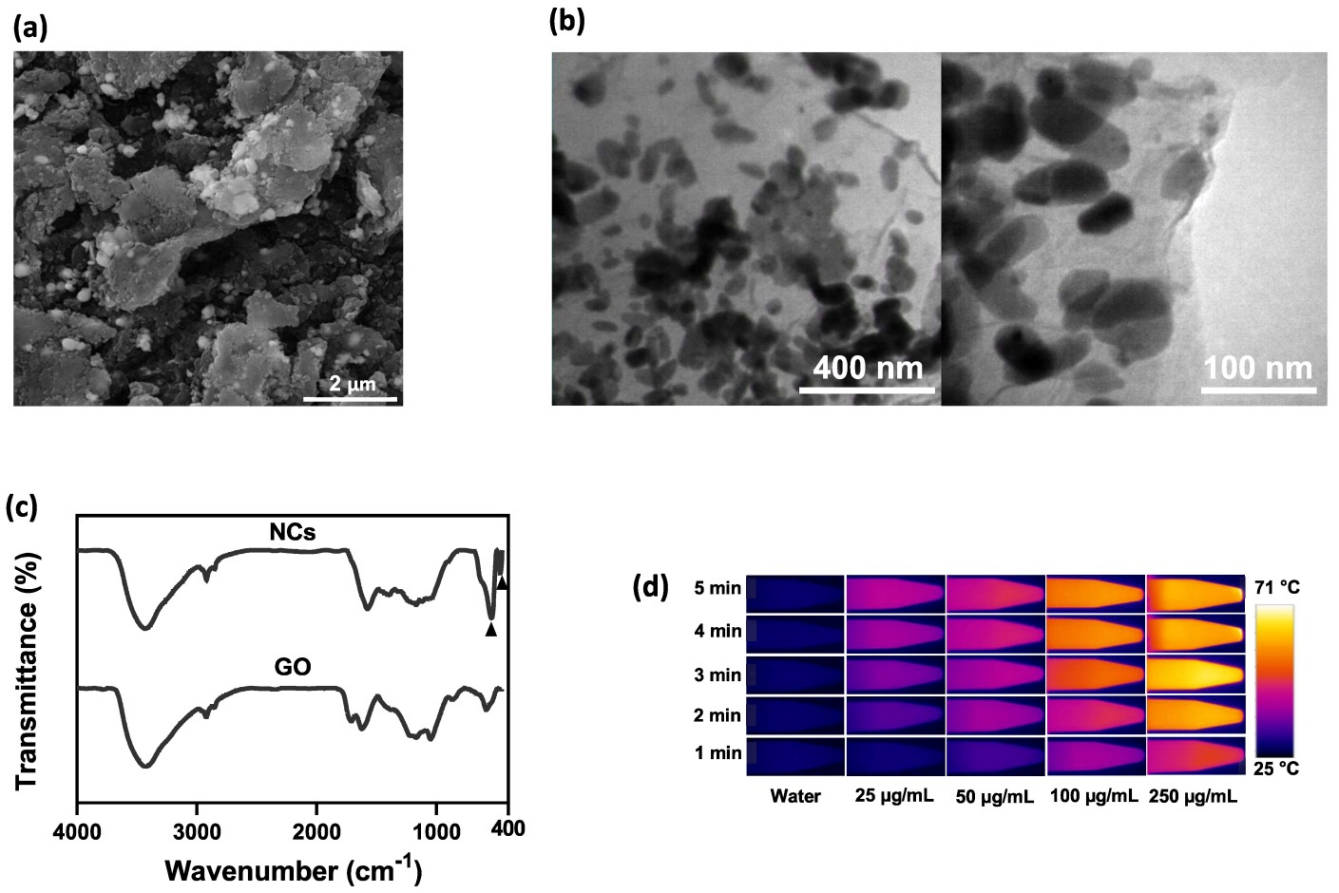

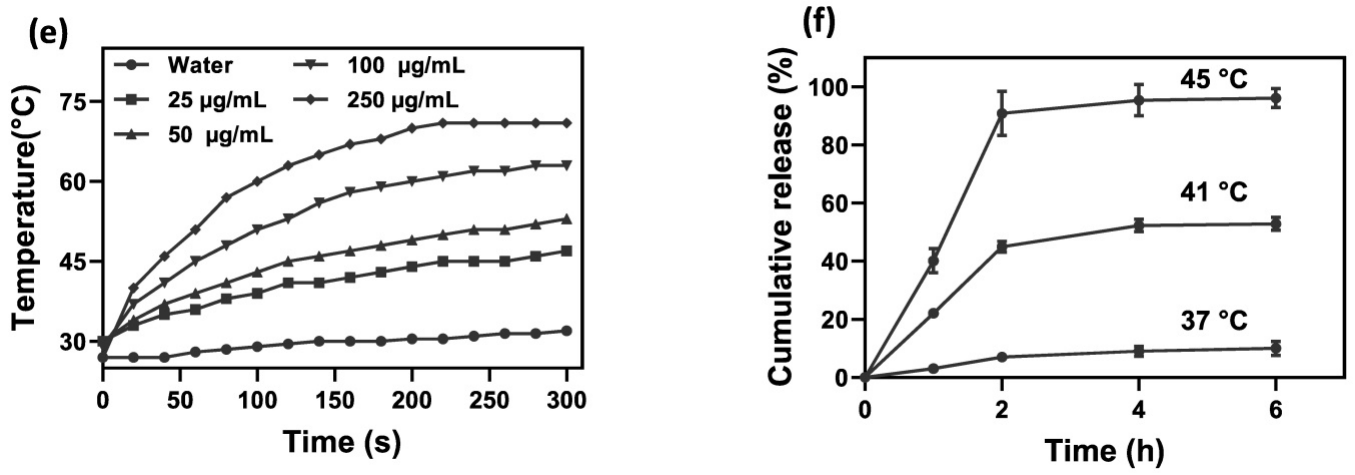
(a) SEM and (b) TEM images of NCs. (c) FTIR spectra of GO and GO-SPIO-Au. (d) Representative IR thermal images and (e) temperature change profiles of the aqueous NC solutions during NIR laser irradiation (1.8 W/cm2). (f) Cumulative DOX release from aqueous NC solutions (pH=7) at different temperatures.

Figure 1 shows a schematic diagram of the synthesis of NIR irradiation-responsive NCs. After oxidizing the natural graphite to obtain GO, SPIOs were attached on the surface of GO nanosheets through a hydrothermal reaction, followed by the reduction of HAuCl_4_ with trisodium citrate dehydrate to form GO-SPIO-Au. Next, GO-SPIO-Au NCs were coated with the drug-loaded PCM and modified with an alginate hydrogel polymer. The PCM shows a surfactant-like behavior, attached to the surface of GO-SPIO-Au through long hydrophobic tails. Morphological investigation by FE-SEM indicated that the NCs have a multilayer structure with the aggregation of nanoparticles manifested as bright spots on the surface of GO sheets (Figure 2a). TEM revealed that nanoparticles with an average diameter of 40 nm are successfully deposited on the GO sheets (Figure 2b). The FTIR spectra of GO-SPIO-Au showed two absorption peaks at around 539 and 467 cm^-1^, which can be attributed to stretching vibrations of the Fe-O band of SPIO NPs, indicating the successful decoration of GO nanosheets with SPIO NPs (Figure 2c). The XRD analysis characterized the crystalline structure of the NCs, showing diffraction peaks corresponding to presence of Au and SPIO NPs (Supplementary Information, Figure S1). The magnetic hysteresis curve of the NCs at room temperature also confirmed the magnetization of the NCs due to presence of SPIO (Figure S2). Furthermore, the UV-vis spectra of the NCs corroborated the presence of AuNPs by showing an absorption peak around 533 nm (Figure S3).

To assess the photothermal effect of NCs, varying concentrations of NCs were exposed to NIR irradiation and the temperature variation was monitored in real-time using an IR thermal camera. Compared to pure water, NCs solutions showed a significantly increased heating rate (P value < 0.5) in a concentration-dependent manner (Figure 2d, e). After 5 min NIR irradiation, the water temperature increased by 5 °C, whereas NC solutions at concentrations of 25, 50, 100, and 250 µg/mL had markedly higher temperature rises of 17, 23, 34, and 44 °C, respectively. Based on the thermometry results and the formulas provided in the Supplementary Information, the photothermal conversion efficiency of the NCs was calculated as 59% 51. Furthermore, as shown in Figure S5, NCs and GO suspensions at a given GO concentration showed a comparable temperature rise rate under NIR irradiation, proving that AuNPs have no notable photothermal effect. It has been widely reported that AuNPs with different shapes such as rods, cages and stars are tuned in the NIR region and can therefore be used as NIR photothermal agents. However, spherical AuNPs embedded in the structure of NCs are tuned with light in the visible region. Indeed, the absorbance of AuNPs under NIR laser irradiation is negligible and cannot contribute to heat generation 52. Hence, NCs are able to effectively absorb NIR light and convert it into heat, allowing photothermal ablation of tumor cells.

To confirm the thermosensitive drug release of NCs, the *in vitro* release of DOX from NCs was studied by external heating in a water bath. Figure 2f shows that NCs have a slow-release rate at 37 °C (below *T*_m_), with the cumulative DOX release reaching only 13% of the total amount of loaded DOX after 24 h, a fraction which can be classified as uncontrolled drug release. Hence, the PCM is able to retain the drug molecules on the surface of the NCs below its melting point. In contrast, under external heating above the *T*_m,_ the NCs exhibited a dramatically faster DOX release rate resulting from PCM transformation from a solid to a liquid state. After 24 h, the cumulative DOX release from the NCs reached approximately 57 and 98% for 41 and 45 °C, respectively. The amount of drug release from NCs can be thus be regulated by adjusting the temperature.

### CT/MR imaging

**Figure 3:**
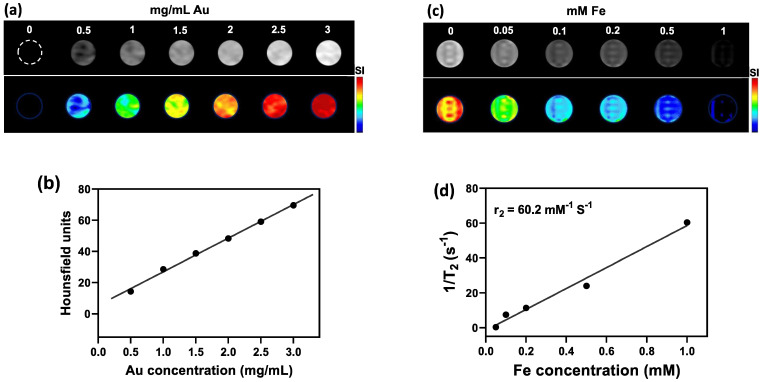

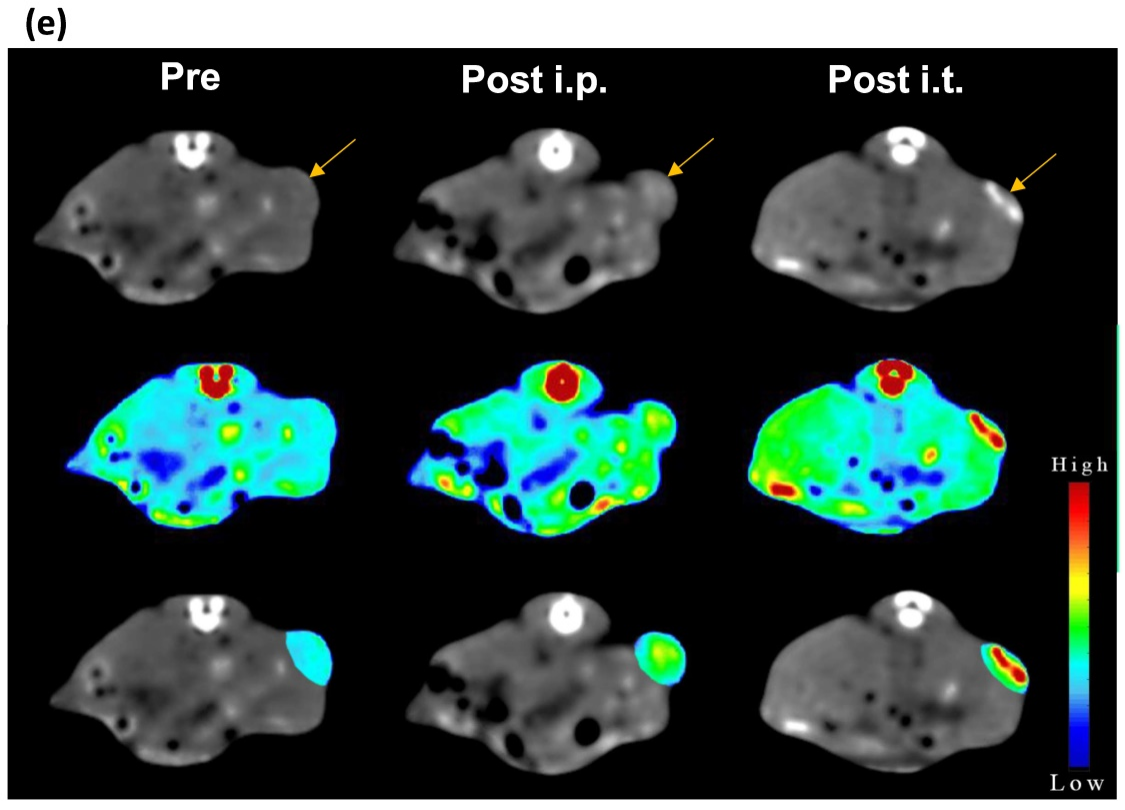

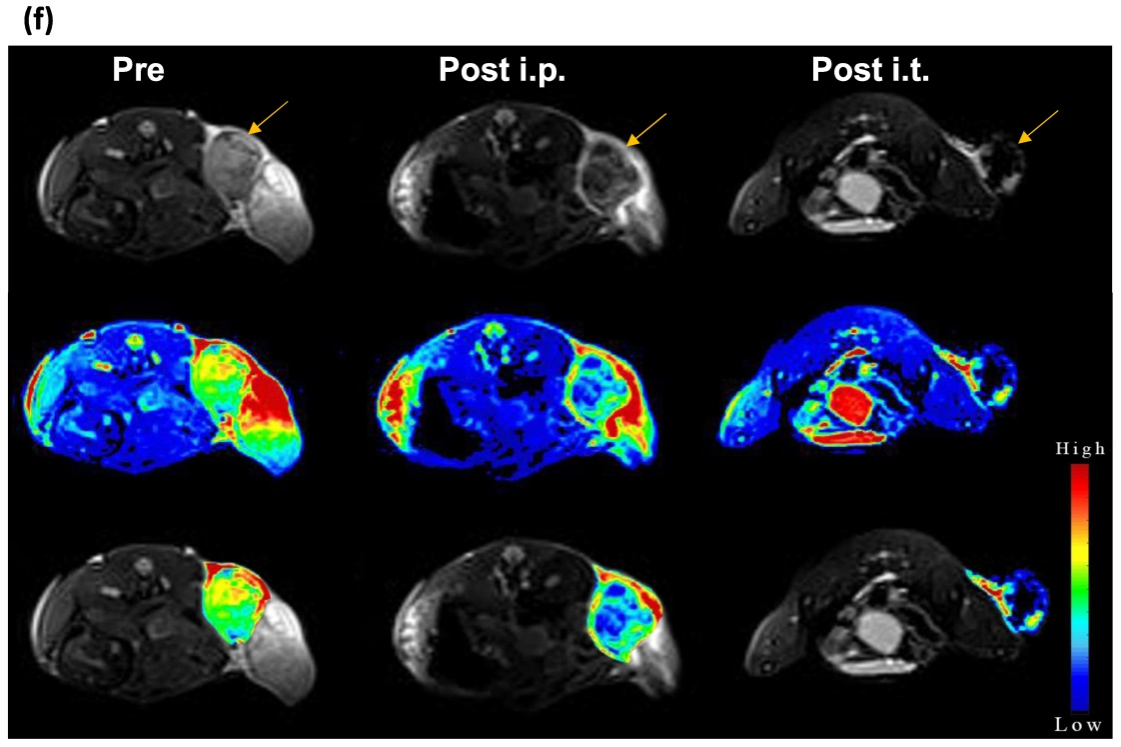
(a) CT images and (b) HU values of NC solutions as function of Au concentration. (c) T2-weighted MR images and (d) transverse relaxation rates (1/T2) of NC solutions as function of Fe concentration. (e) CT and (f) MR images of CT26 tumor-bearing mice before, at 24 h after i.p., and immediately after i.t. injection of NCs (upper row: original images, middle row: signal intensity maps, lower row: merged images). Arrow indicates tumor location.

Owing to their high atomic number (Z=79) that provides a high X-ray absorption cross-section, AuNPs embedded within the NCs could be detected with CT imaging (Figure 3a). Quantification of signal using Hounsfield Units (HU) showed a linear increase of CT contrast with increasing Au concentration (Figure 3b). As for the SPIO component of NCs, T2-weighted MR imaging at 3T MR revealed a concentration-dependent hypointense contrast (Figure 3c), with a transverse relaxivity (*r*_2_) of 60.15 mM^-1^ s^-1^ (Figure 3d). The obtained value of r_2_ is in the range of that previously reported for magnetic nanoparticles 53. For example, Guo *et al.* developed thermosensitive magnetoliposomes for light/magnetic hyperthermia-triggered drug delivery having an r_2_ of 60.06 mM^-1^ s^-1^ which is comparable to our NCs 54. Similar to the aim of this study, Xie *et al.*, developed a multifunctional nanoplatform by constructing core/shell structures of doxorubicin-loaded Fe_3_O_4_@molybdenum disulfide for MRI-guided chemo-photothermal therapy, with an r_2_ value of 48.86 mM^-1^ s^1^ 55.

Next, we studied the potential of NCs to serve as tumor imaging probes *in vivo*. A striking contrast enhancement was observed on CT imaging, with HU values increasing from 87.29±5.4 before injection to 111.22±18.2 HU and 139.48±38.6 HU 24 h post i.p. (30 mg Au/kg) or immediately post i.t. (3 mg Au/mL, 30 µL) injection, respectively (Figure 3e). MR images acquired post injection of NCs revealed a characteristic hypointense contrast, with signal intensity decreasing from 86.5±7.6 before injection to 56.3±9 and 27.12±32.6, 24 h post i.p. injection (40 mg Fe/kg) and immediately after i.t. injection (4 mg Fe/mL, 30 µL), respectively (Figure 3f). These results indicate that the NCs can be used as dual CT/MR contrast agents.

### *In vitro* hemolytic and cytotoxicity assessment

**Figure 4:**
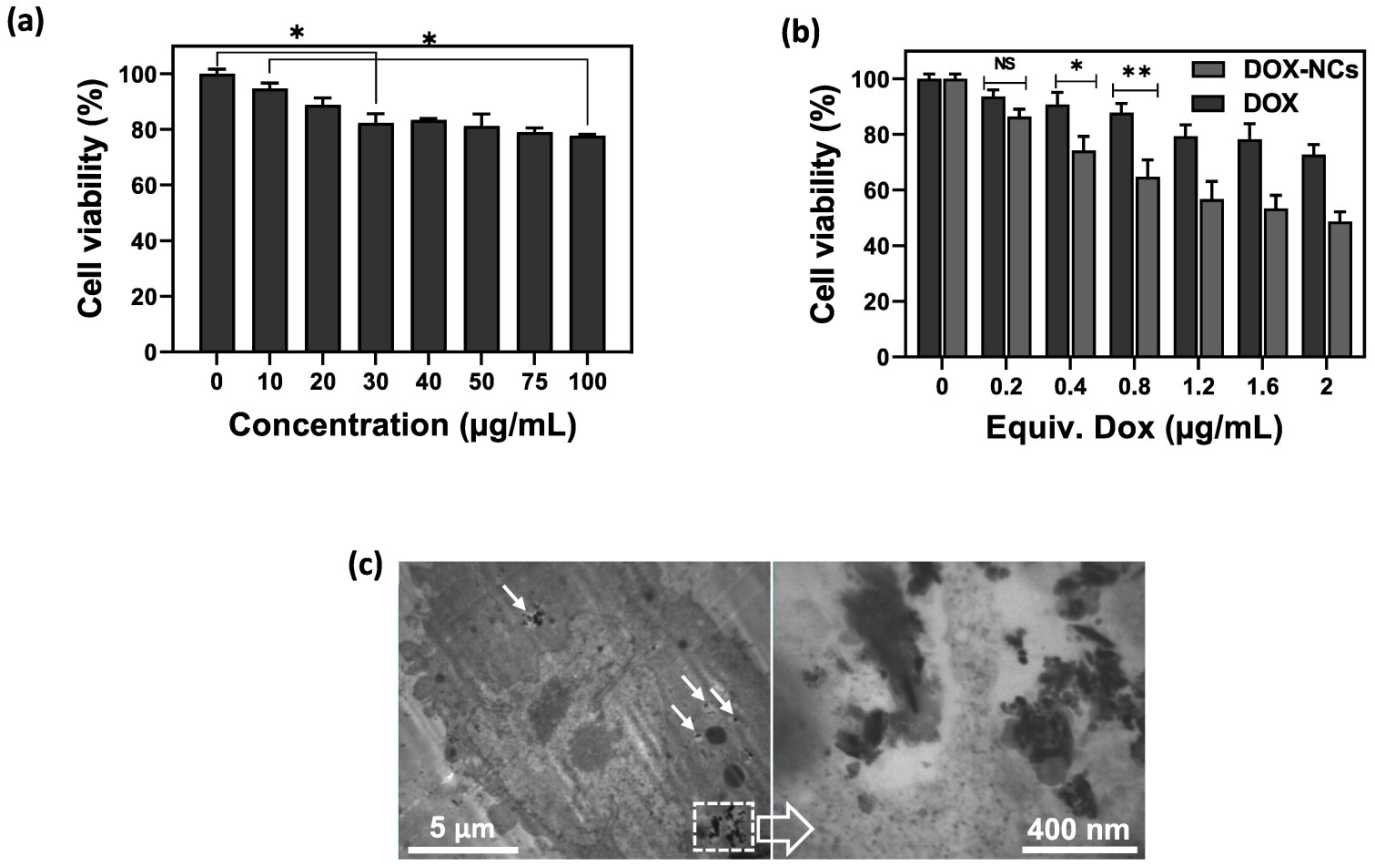
(a) Viability of CT26 cells after 6 h incubation with various concentrations of GO-SPIO-Au-TD-Alg. (b) Viability of CT26 cells after 6 h incubation various concentrations of free DOX and GO-SPIO-Au-DOX-TD-Alg (DOX-NC). (c) TEM image of CT26 cells after 6 h incubation with 50 µg/mL NC shows intracellular NC accumulation (arrows). NS: not significant, *P<0.05, **P<0.01.

The hemolytic properties of the NCs were assessed with a hemolytic assay (Figure S6). As compared to the positive water control that leads to hemolysis, NCs displayed no apparent hemolysis effect, similar to the negative PBS control. The viability of CT26 cells after incubation with GO-SPIO-Au-TD-Alg at different concentrations (0-100 µg/mL) was measured using an MTT assay to evaluate the biocompatibility of drug-free NCs. A gradual increase in cytotoxicity was noted with increasing concentration (Figure 4a), with the cell viability reduced to ~78% at the highest concentration of 100 µg/mL. To compare the cytotoxicity of free drug and drug-loaded NCs, cells were incubated with DOX and DOX-NCs for 6 h. The viability of DOX-treated CT26 cells decreased from 94% to 73% for 0.2 and 2 µg/mL, respectively, whereas the viability of DOX-NC-treated cells decreased from 86 to 49% at the same DOX concentration range (Figure 4b). The lower viability of cells treated with DOX-NCs compared to cells treated with free DOX may result from a more efficient cellular uptake and drug retention using the nanoparticles. TEM demonstrated that the NCs were able to pass through the cell membrane and efficiently accumulated inside cells (Figure 4c).

### *In vitro* combined thermo-chemotherapy

**Figure 5:**
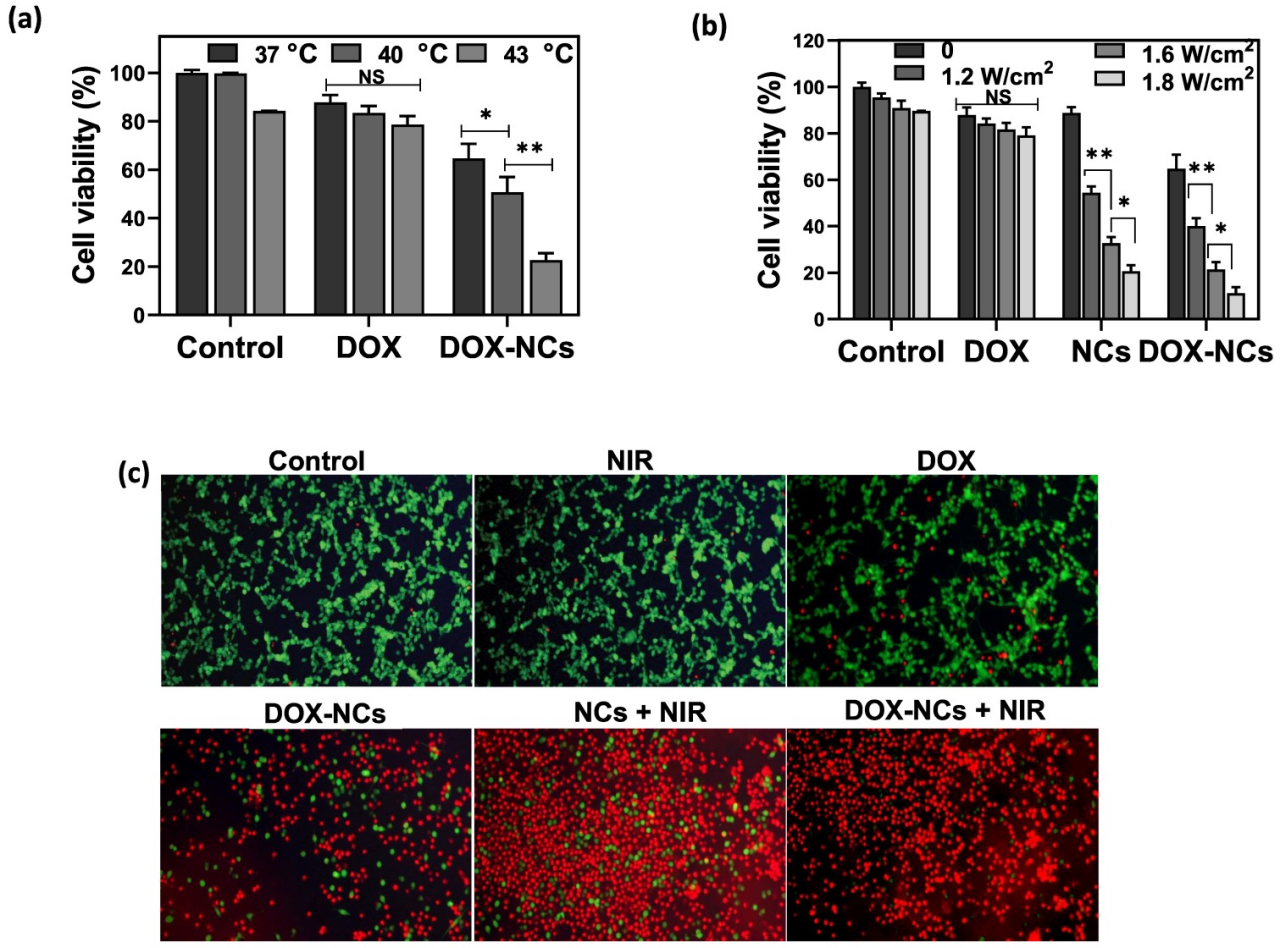
(a) Viability of CT26 cells pretreated with DOX and DOX-NC at equivalent DOX concentrations of 0.8 µg/mL and exposed to different temperatures for 1 h. (b) Viability of CT26 cells after 6 h incubation with DOX, GO-SPIO-Au-TD-Alg (NCs) and GO-SPIO-Au-DOX-TD-Alg (DOX-NC), followed by NIR irradiation at different power densities for 5 min. (C) Live/dead staining of CT26 cells after 6 h incubation with various formulations (NC concentration = 20 µg/mL; DOX concentration = 0.8 µg/mL; NIR irradiation= 1.8 W/cm2 for 5 min). Viable cells stain green (FDA) and dead cells red (PI). NS: not significant, *P<0.05, **P<0.01.

Loading of DOX within TD acting as a PCM enable an accelerated payload release from the NCs in response to heating. To test the thermosensitive potential of the NCs, cells were treated with DOX and DOX-NCs at an equivalent DOX concentration of 0.8 µg/mL and then exposed to different temperatures of 37, 40, and 43 °C for 1 h (Figure 5a). Cells treated with DOX showed a gradual decrease in viability when exposed at higher temperatures. In contrast, upon heat exposure, the viability of cells treated with DOX-NCs decreased radically from 65% (37 °C) to 51% (40 °C) and 23% (43 °C). Since NCs can generate heat upon NIR light irradiation owing to the presence of GO as a photothermal agent, we next investigated the effect of NIR irradiation with different power densities on the tumor cell killing efficiency of DOX, NCs, and DOX-NCs. Figure 5b shows that NIR irradiation alone does not induce cell death, with a cell viability >90% even at the highest power density of 1.8 W/cm^2^. There was also no change in the viability of cells treated with NIR irradiation when incubated with free DOX. Under the same NIR irradiation conditions, cells treated with thermo-chemotherapy (DOX-NC + NIR) showed a significantly higher cytotoxicity (P<0.05) than cells receiving PTT alone (NCs + NIR). The therapeutic effects of the various treatments were also investigated using a live/dead staining assay in which viable cells are stained green with FDA and dead cells are stained red with PI. As shown in Figure 5c, the combination of DOX-NCs and NIR induces an enhanced cell damage, indicating the synergistic effects of thermo-chemotherapy.

### Detection of necrosis and apoptosis

**Figure 6:**
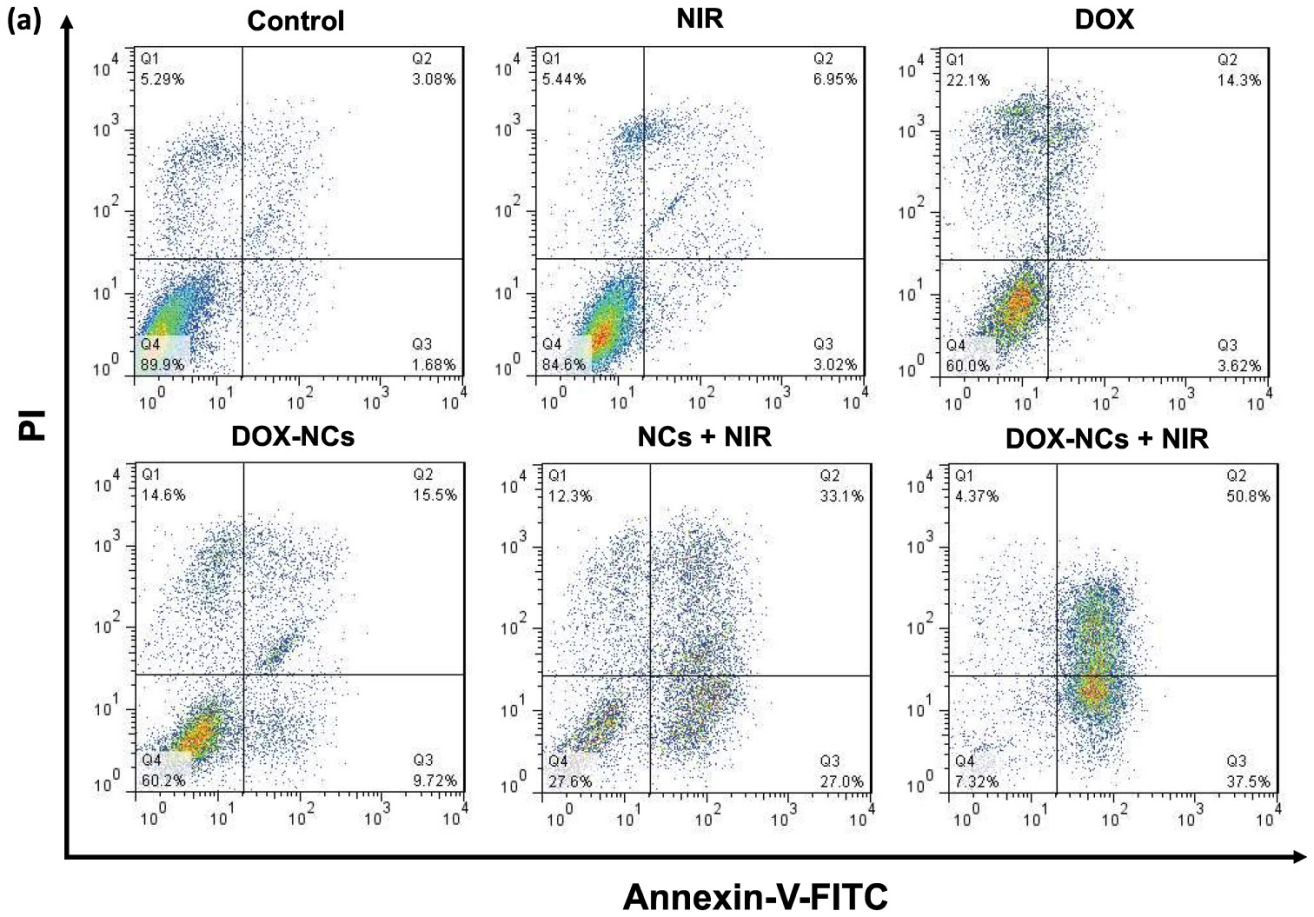

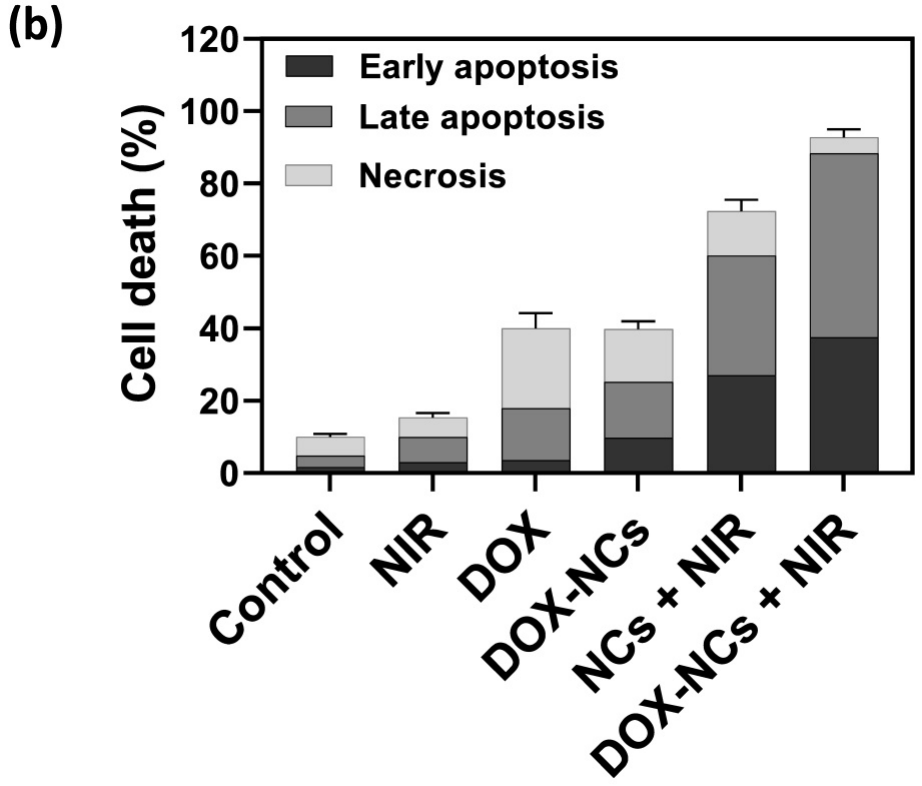
(a) Flow cytometry of CT26 cell death using Annexin-V-FITC/PI staining after 6 h incubation with various formulations (NC concentration = 20 µg/mL; DOX concentration = 0.8 µg/mL; NIR irradiation= 1.8 W/cm2 for 5 min). (b) Percentage apoptosis and late apoptosis/necrosis for the different treatments.

Necrosis and apoptosis are the two main mechanisms of cancer cell death in response to different treatments. Necrosis is accompanied by inflammatory and immunogenic responses around the cells due to the release of intracellular constituents. In contrast, apoptosis as a programmed cell death mechanism has no detrimental effect on surrounding healthy cells and is therefore preferable when treating tumors 56. While early apoptotic cells preserve their plasma membrane integrity to prevent the potentially harmful cellular contents from leakage, the plasma membrane in late apoptotic cells becomes permeable, resulting in release of intracellular molecules that provoke inflammatory responses 57. To quantitatively determine the amount of necrosis and apoptosis following cancer cell treatment, flow cytometry was performed with Annexin-V-FITC/PI staining (Figure 6a, b). In comparison to the untreated control, almost no apoptosis was observed in the groups receiving NIR irradiation (1.8 W/cm^2^, 5 min) and DOX (0.8 µg/mL). The rate of apoptosis in CT26 cells increased from 3.6% for DOX to 9.7% for DOX-NCs, indicating that chemotherapy with the NCs is more effective in inducing apoptosis than the free drug. Interestingly, there was a notable increase in the proportion of cells undergoing apoptosis upon NC-mediated PTT and thermo-chemotherapy, with apoptosis levels of 27 and 37.5%, respectively. These results demonstrate that the synergistic tumor killing effects of DOX-NCs and NIR light irradiation are a result from enhanced apoptosis.

### *In vivo* anti-tumor therapeutic efficacy

**Figure 7:**
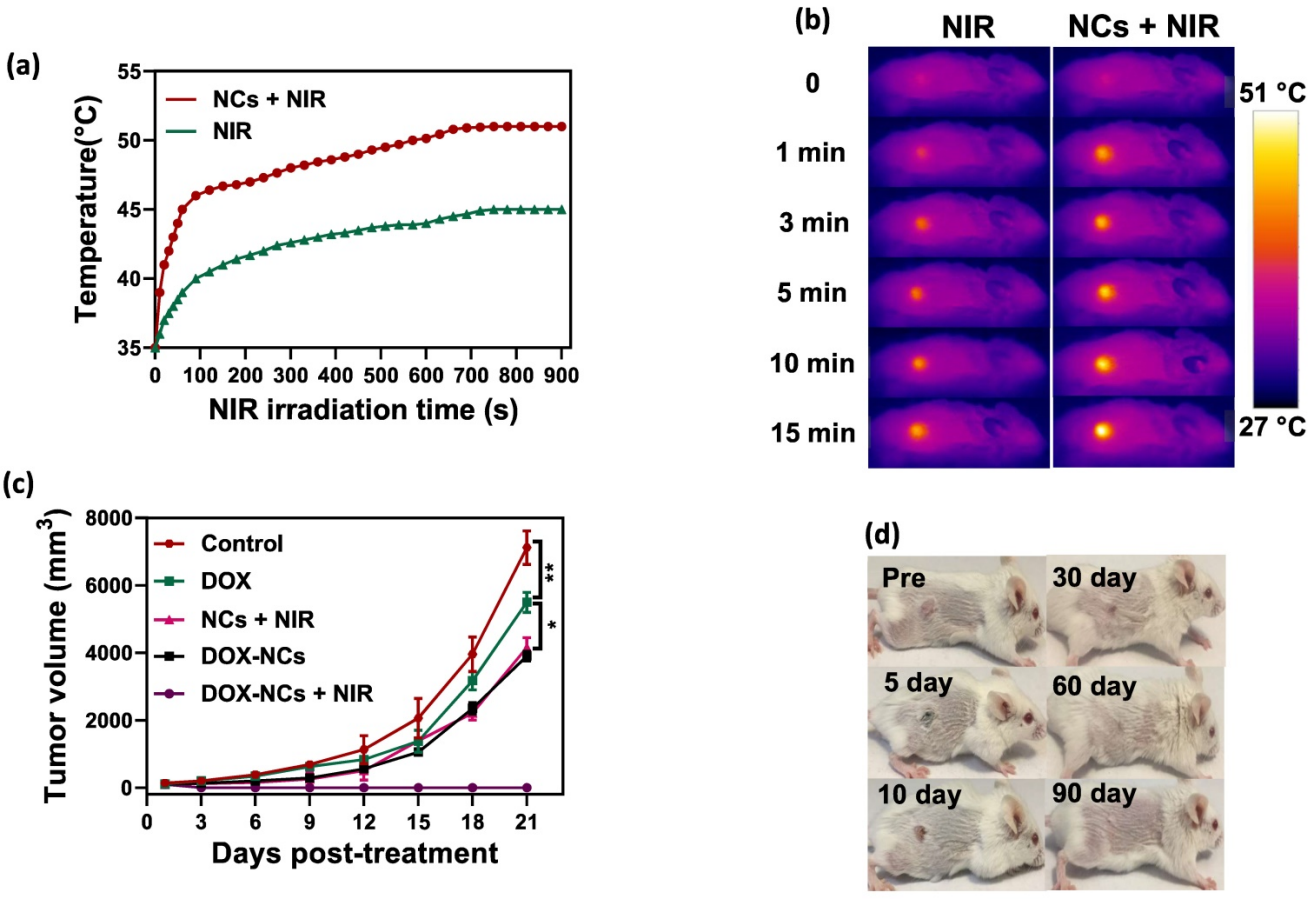

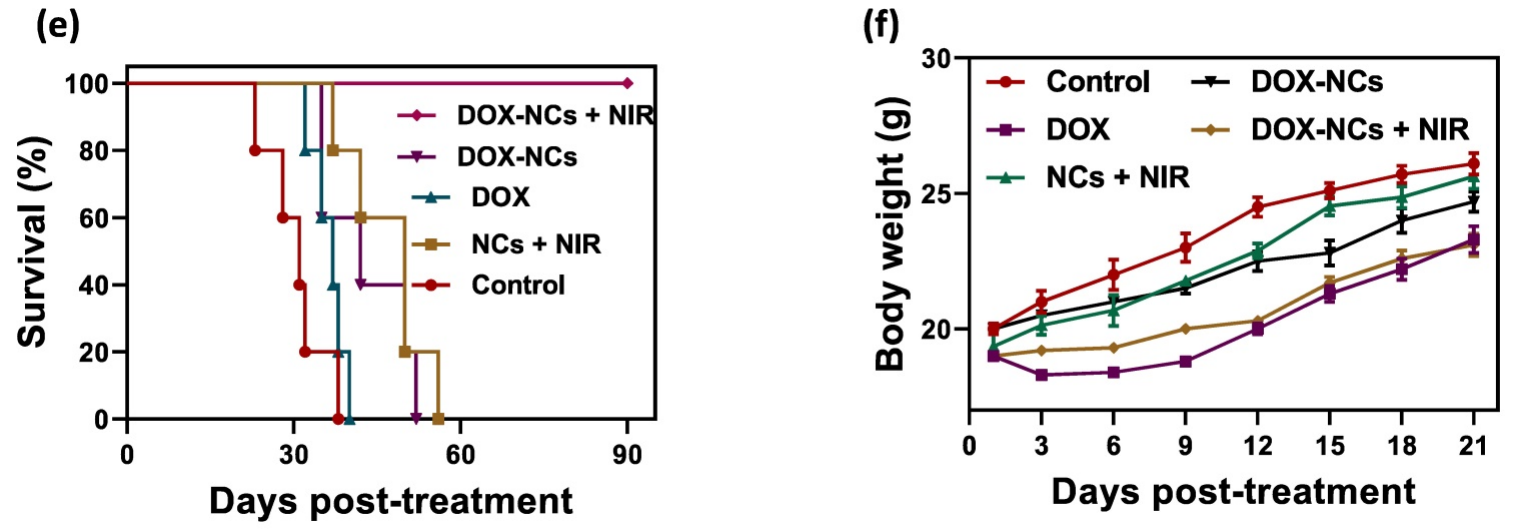
(a) Temperature rise profiles for CT26 tumors and (b) representative IR thermal images of CT26 tumor-bearing mice with and without NC injection under NIR irradiation (0.7 W/cm2). (c) Tumor growth curves for the various treatments. *P<0.01, ** P<0.001. NIR irradiation was performed 24 h post i.p. injection. (d) Representative photographs of tumor-bearing mice in the group receiving thermo-chemotherapy for 90 days post-treatment. (e) Survival curves of tumor-bearing mice for the various treatments. (f) Change in body weight of tumor-bearing mice for the various treatments.

To demonstrate the PTT effect of NCs *in vivo*, the temperature profile of the tumor with and without i.p. injection of NCs was recorded in real-time using an IR thermal camera. Upon NIR irradiation, the tumors 24 h pre-treated with NCs, rapidly rises in temperature from 35 to 51 °C after 15 min. In contrast, without NC injection the tumor only reached 45 °C under the same laser exposure conditions (Figure 7a, b). Figure 7c shows tumor growth curves for the 5 different treatment regimens over 21 days post-treatment. Systemic i.p. injection of DOX slightly delayed the tumor growth rate compared to the untreated control, with a tumor growth inhibition (TGI) rate of 22.7%. In contrast, the same dose of DOX conjugated to the NCs resulted in a TGI = 45.2%. Mice treated with naked NCs (no DOX) + NIR irradiation had a TGI = 41.5%, which is comparable to the group receiving DOX-NCs. To synergistically combine PTT-induced heat generation with a heat-triggered DOX release from NCs, mice were i.p. injected with DOX-NCs and then subjected to NIR irradiation after 24 h. The tumors in this thermo-chemotherapy group were completely eradicated without showing evidence of regrowth over a 90-day follow-up period. Tumor-bearing mice were also monitored for their survival up to 90 days post-treatment (Figure 7e). Although mice treated with DOX-NCs and NCs + NIR showed a statistically significant prolongation of survival rate as compared to non-treated animals, all mice in these two groups had died within two months. Animal body weight was also recorded as a parameter for systemic toxicity assessment (Figure 7f). Injection of DOX alone resulted in a decrease in body weight within several days post-injection. However, no such reduction in body weight was observed for the other treatment groups, indicating that injection of DOX-NCs in combination with NIR irradiation is well-tolerated by the animals.

### Histopathological results

**Figure 8:**
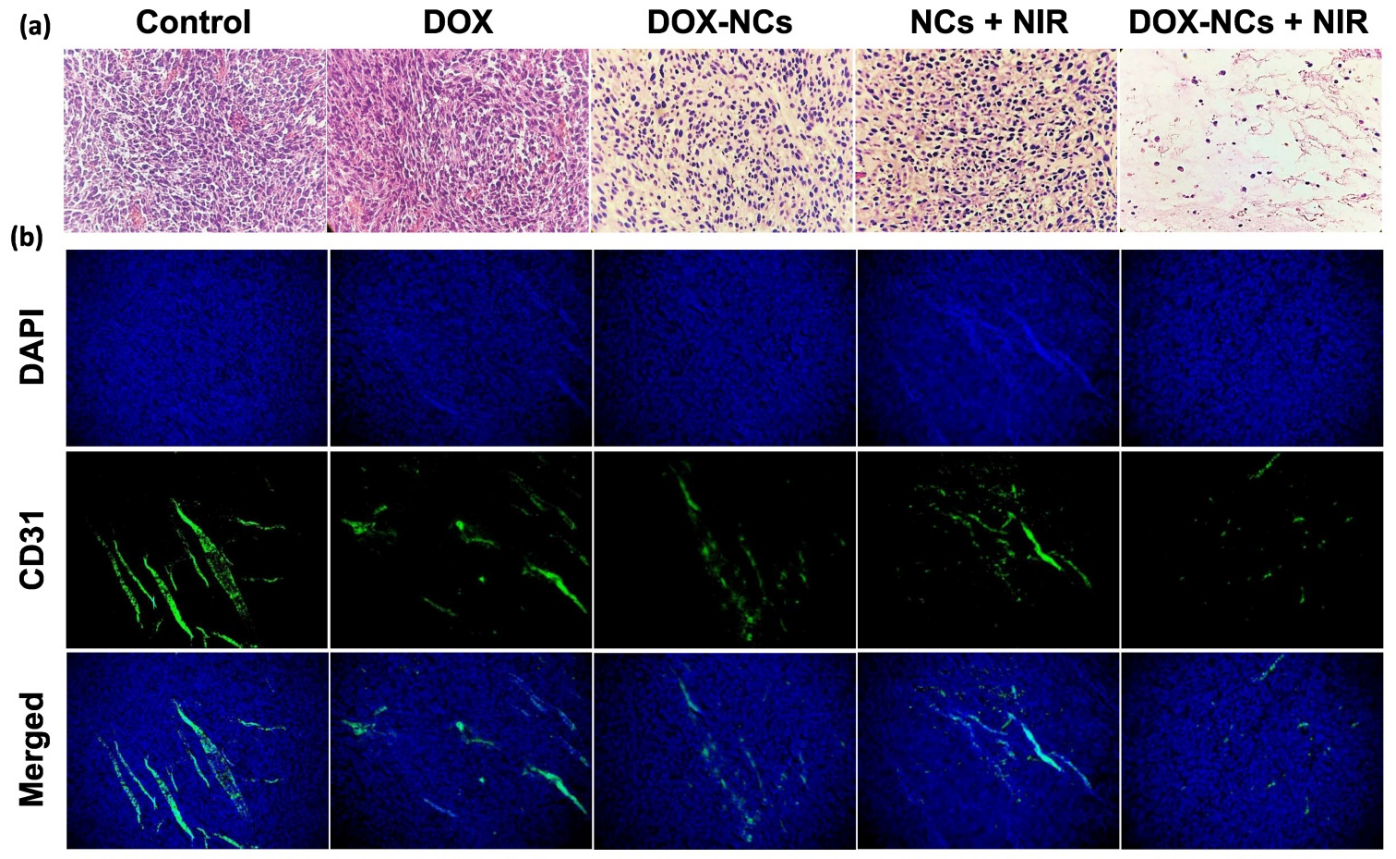

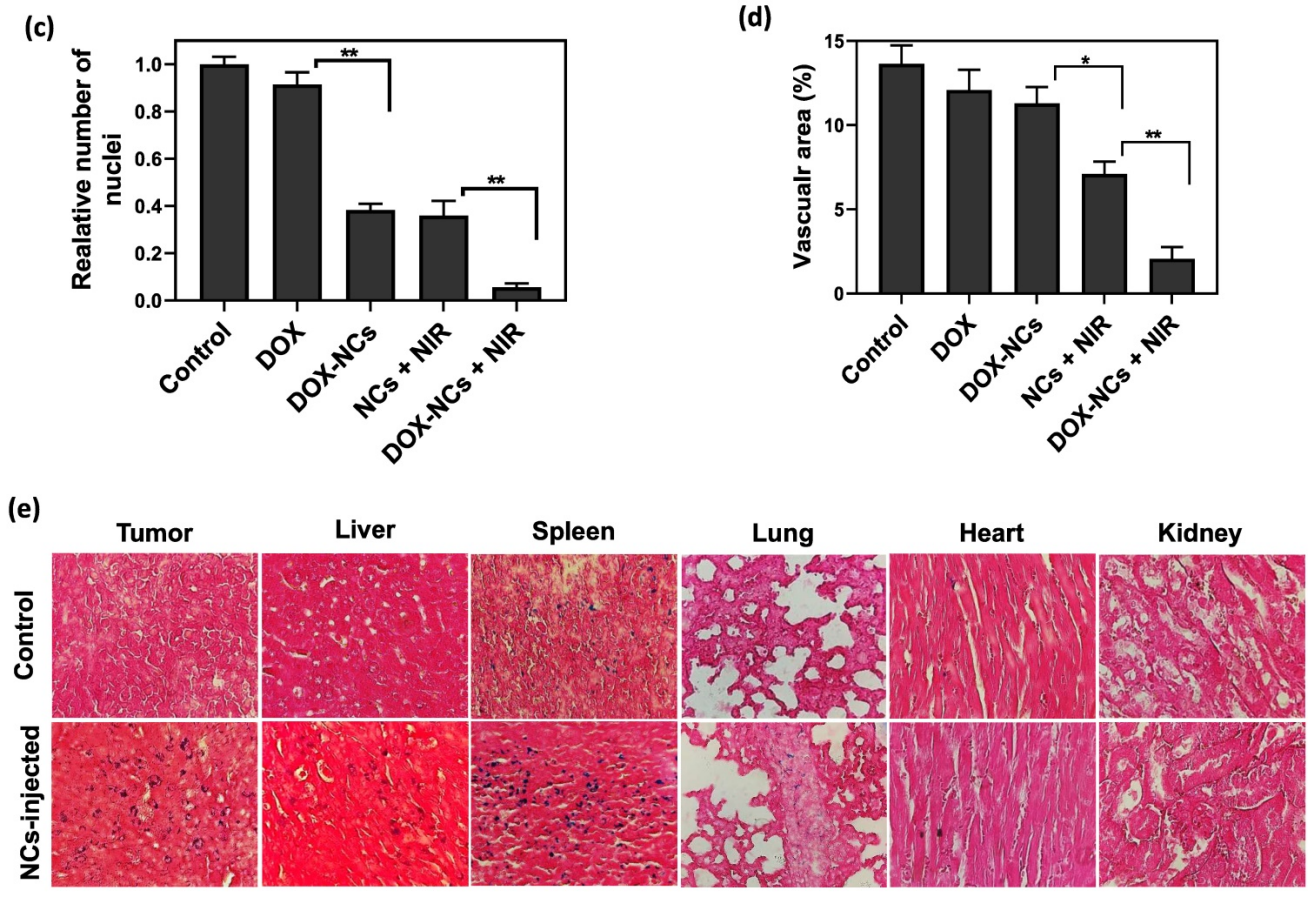
(a) Representative H&E-stained histological images of tumor tissue and (b) IHC analysis of tumor vasculature for the different treatment groups at 3 days post-treatment. Blood vessels are shown in green (FITC-labeled anti-CD31) and nuclei are shown as blue (DAPI). Digitized (c) H&E-stained images and (d) anti-CD31-stained images for quantification of tumor vascular density using ImageJ software. *P<0.01, **P<0.001. (e) Representative Prussian Blue-stained tissue sections of the tumor and other organs at 24 h post i.p. injection of NCs.

Tumor specimens were harvested 3 days post-treatment for histopathological examination. H&E-staining showed that NC-mediated thermo-chemotherapy exhibited the highest tumor cell removal efficiency and left extensive necrotic area in the tumor (figure 8a). Compared to the untreated control, the DOX-treated tumor showed no significant decrease in cell density, whereas the cell density was reduced by 62.7, 64%, and 95% in the DOX-NCs, NCs + NIR, and DOX-NCs + NIR groups, respectively (Figure 8c). Anti-CD31 immunohistochemical staining of vascular endothelial cells demonstrated that the tumor in the control group had an overall intact and rich vascularity, whereas the different treatment groups showed damaged blood vessel structures with a decreased density (Figure 8b). As expected, the combination of DOX-NC + NIR irradiation yielded the highest vascular damage efficiency, being 85% compared to that of the untreated group, further demonstrating the enhanced therapeutic efficiency of NC-mediated thermo-chemotherapy (Figure 8d). PB staining of tumor tissue at 24 h post injection of NCs (Figure 8e) revealed the presence of iron-(SPIO) positive cells, confirming efficient tumor localization of NCs following systemic injection, consistent with the *in vivo* imaging results. Since the size of the NCs is larger than the renal clearance threshold (~6 nm), one can expect that the particles are removed predominantly by the reticuloendothelial system 58. Indeed, PB-positive cells were primarily found in the liver and spleen, with few or none in the lung, kidney, and heart. Consistent with PB staining, the biodistribution analysis using ICP-MS confirmed effective accumulation of the NCs in tumor with nearly 3.77% of the injected dose per gram of tumor (Figure S7). More importantly, the results of ICP-MS are in accordance with previous studies reporting that nanoparticles larger than the renal clearance threshold (~6 nm) are predominantly taken up by the reticuloendothelial system in the liver and spleen 59. Therefore, further studies on the potential long-term toxic effects of nanoparticles in these organs are warranted.

## Conclusions

In summary, we developed a novel 2D nanotheranostic platform by anchoring SPIOs and AuNPs on GO nanosheets, adapted with a PCM material for stimulus-responsive drug release, with inclusion of Alg hydrogel to assure biocompatibility. The strong NIR absorbance of GO, MRI-contrast enhancement of SPIOs, X-ray attenuation of AuNPs, and the thermosensitive feature of PCM-mediated drug release make this platform ideally suited for synergistic thermo-chemotherapy in a controllable drug-release fashion, and which can be visualized with CT/MR bimodal imaging.

## Supplementary Material

Supplementary figures.
