# Supplementary material for: A 2D nanotheranostic platform based on graphene oxide and phase-change materials for bimodal CT/MR imaging, NIR-activated drug release, and synergistic thermo-chemotherapy
Source: Nanotheranostics. 2022 May 24;6(4):350–64. doi: 10.7150/ntno.64790 (PMC9194590; doi:10.7150/ntno.64790)
Supplement: Supplementary file 1 — Supplementary figures. [file ntnov06p0350s1.pdf]

Supplementary Information

A 2D nanotheranostic platform based on graphene oxide and phase-change materials for bimodal CT/MR imaging, NIR-activated drug release, and synergistic thermo-chemotherapy

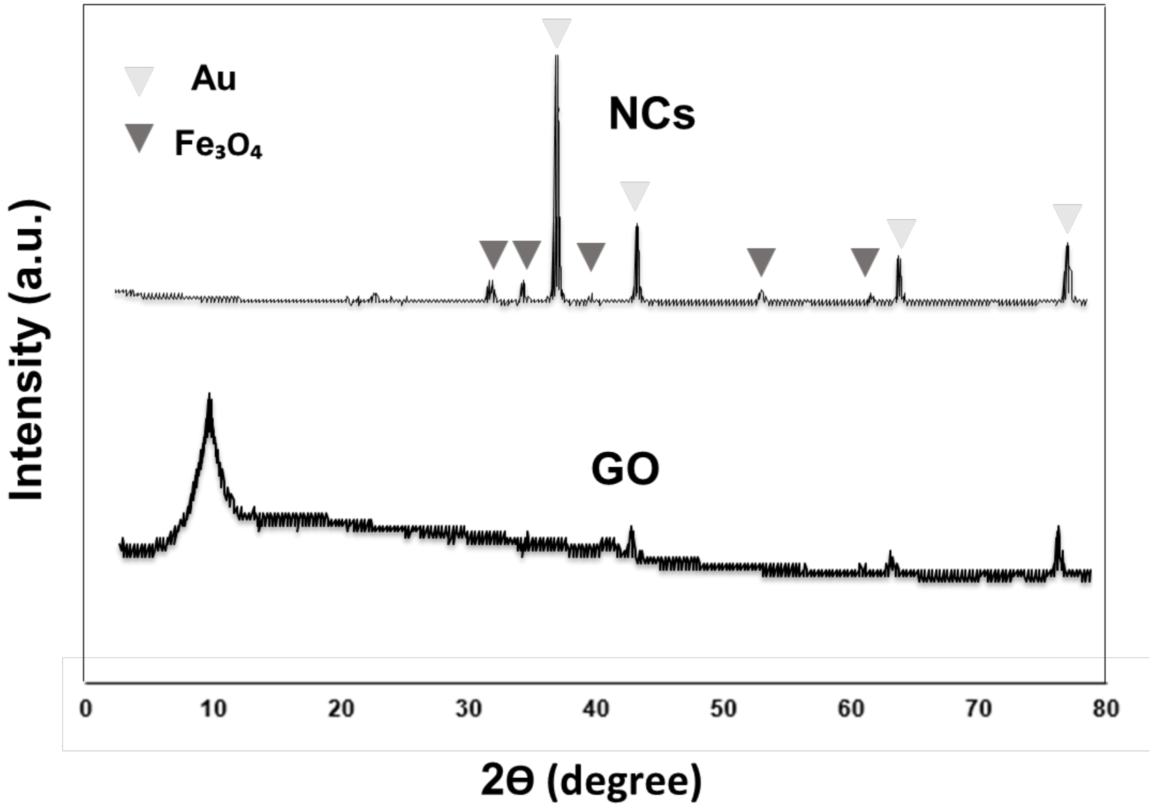

Figure S.1. The XRD patterns of GO and NCs, showing the characteristic peaks at 2θ values of 32.82° (220), 35.40° (311), 43.69° (400), 53.72° (422), and 62.26° (440), which are attributed to the cubic inverse spinel structure of SPIO. The diffraction peaks at 2θ values of 37.92° (111), 44.16° (200), 64.36° (220) and 77.62° (322) also correspond to the cubic crystals of gold.

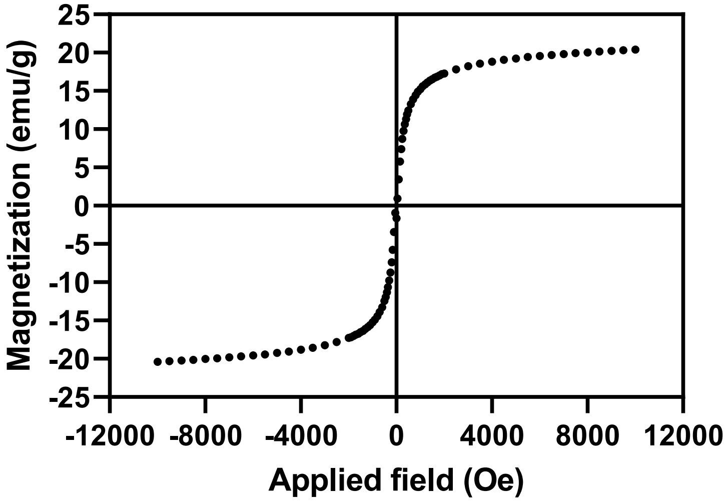

Figure S.2. The magnetometry measurement of room-temperature magnetic hysteresis of the NCs. The saturation magnetization was measured to be 13.6 emu/g.

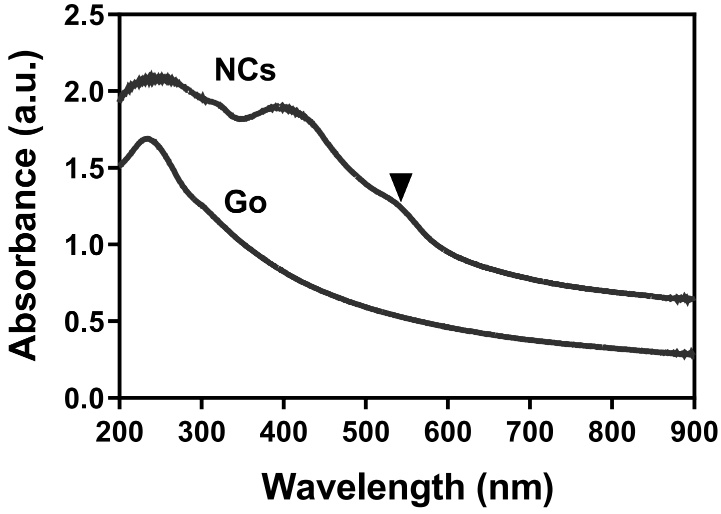

Figure S.3. The UV-vis spectra of NCs shows a red shift compared to GO, along with a peak around 533 nm corresponding to AuNPs.

Calculation of photothermal conversion efficiency

The photothermal conversion efficiency (η) of NCs was calculated according to the following formulas [1, 2]:

$$\eta = \frac{hS\,(T_{max} - T_{sur}) - Q_{dis}}{I(1-10^{-A_{808}})} \qquad (1)$$

where h is the heat transfer coefficient, S is the surface area of the container, T_{max} and T_{sur} are the maximum equilibrium temperature and the ambient temperature, I is the laser power, A_{808} is the absorbance of NCs at 808 nm, and Q_{dis} is heat dissipation due to light absorbance of the solvent. hS can be calculated according to the following equation:

$$hS = \frac{m_s C_s}{\tau} \qquad (2)$$

where τ is the sample system time constant, and m_s and C_s are the mass and the heat capacity of the solvent (water), respectively. The system equilibration time (τ) was estimated according to previous report by Roper et. al, by applying the negative reciprocal slope of ln (Θ) versus t using temperature versus time data recorded during cooling in Figure S.4.

$$\Theta = \exp(-t/\tau) \qquad (3)$$

Where Θ is a dimensionless driving force temperature and can be calculated as:

$$\Theta = \frac{T_{amb} - T}{T_{amb} - T_{max}}$$

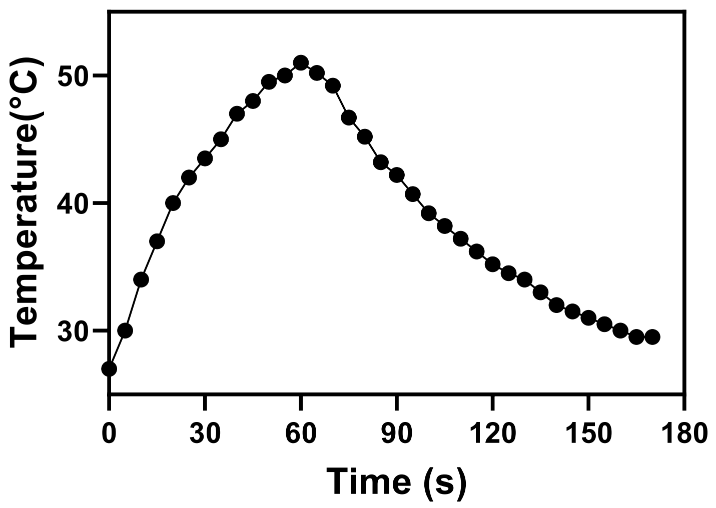

Figure S.4. Temperature changes of the NCs solution (250 μg/mL) during NIR heating (laser on, 1.8 W/cm^2) and colling (laser off) stages.

References:

S1. Bhana S, Lin G, Wang L, Starring H, Mishra SR, Liu G, et al. Near-infrared-absorbing gold nanopopcorns with iron oxide cluster core for magnetically amplified photothermal and photodynamic cancer therapy. ACS applied materials & interfaces. 2015; 7: 11637-47.

2. Hu Y, Wang R, Wang S, Ding L, Li J, Luo Y, et al. Multifunctional Fe3O4@ Au core/shell nanostars: a unique platform for multimode imaging and photothermal therapy of tumors. Scientific Reports. 2016; 6.

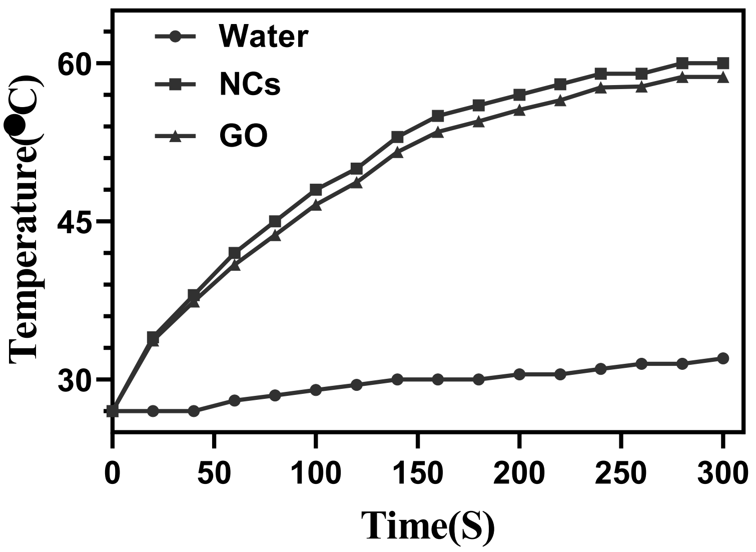

Figure S.5. Temperature change profiles of the aqueous solution of NCs and GO (100 μg/mL per GO) compared to pure water under NIR laser irradiation (1.8 W/cm²).

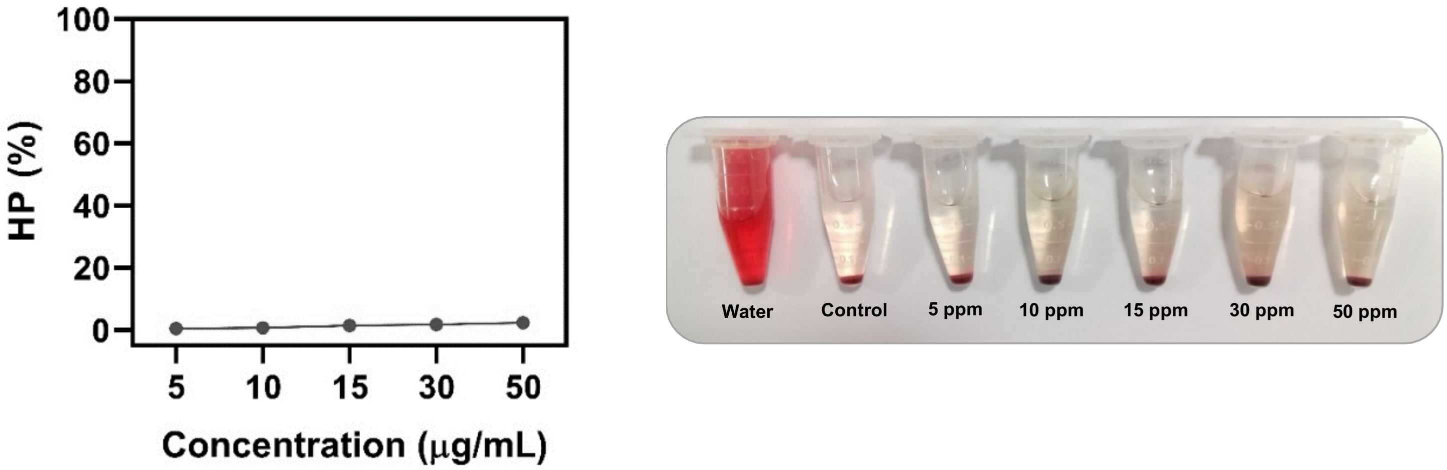

Figure S.6. Hemolysis analysis of the NCs at varying concentrations. Water and PBS served as positive and negative control, respectively.

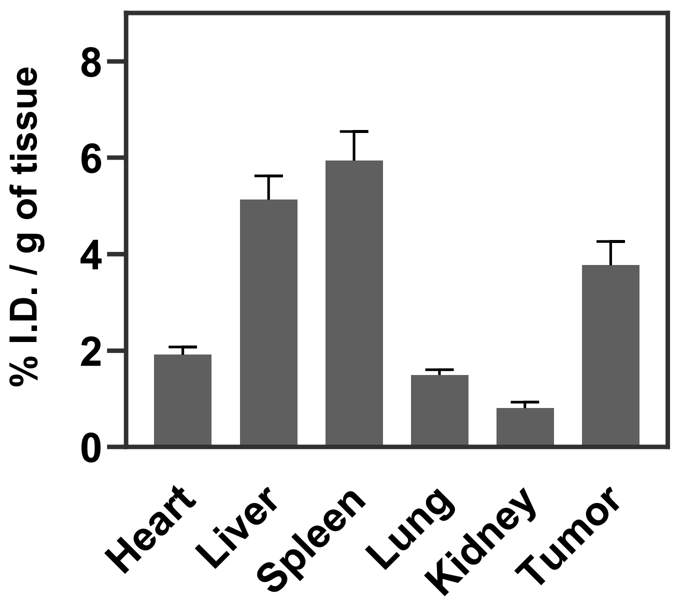

Figure S.7. The biodistribution of NCs in the tumor and major organs at 24 h post i.p. injection through analyzing the Au content of tissue using ICP-MS.
